# Comparison of clinicopathologic characteristics among patients with HBV-positive, HCV-positive and Non-B Non-C hepatocellular carcinoma after hepatectomy: a systematic review and meta-analysis

**DOI:** 10.1186/s12876-023-02925-x

**Published:** 2023-08-23

**Authors:** Bingran Yu, Xuting Zhi, Qiong Li, Tao Li, Zhiqiang Chen

**Affiliations:** 1 Department of Hepatic Surgery, Shanghai Cancer Center, Shanghai Medical College, Fudan University, Shanghai, China; 2 Department of Hepatobiliary Surgery, General Surgery, Qilu Hospital, Cheeloo College of Medicine, Shandong University, 107 West Wen Hua Road, Jinan, 250012 China; 3 Department of Hepatic Surgery, Nanyang Central Hospital, Henan, China; 4 National Engineering Laboratory of Medical Implantable Devices, Key Laboratory for Medical Implantable Devices of Shandong Province, WEGO Holding Company Limited, Weihai, 264210 China

**Keywords:** Non-B non-C hepatocellular carcinoma, Hepatic B virus-related hepatocellular carcinoma, Hepatic C virus-related hepatocellular carcinoma, Clinicopathologic characteristics, Prognosis

## Abstract

**Background:**

The incidence of HBV-negative and HCV-negative hepatocellular carcinoma (NBNC-HCC) is significantly increasing. However, their clinicopathologic features and prognosis remain elucidated. Our study aimed to compare the clinicopathologic characteristics and survival outcomes of NBNC-HCC with hepatitis virus-related HCC.

**Method:**

A literature review was performed in several databases, including PubMed, Embase, Cochrane Library and Web of Science, to identify the studies comparing NBNC-HCC with HBV-positive HCV-negative HCC (B-HCC), HBV-negative HCV-positive (C-HCC) and/or HBV-positive HCV-positive HCC (BC-HCC). The clinicopathologic characteristics and survival outcomes were extracted and pooled to access the difference.

**Results:**

Thirty-two studies with 26,297 patients were included: 5390 patients in NBNC-HCC group, 9873 patients in B-HCC group, 10,848 patients in C-HCC group and 186 patients in BC-HCC group. Patients in NBNC-HCC group were more liable to be diagnosed at higher ages, but with better liver functions and lighter liver cirrhosis. Comparing to B-HCC and C-HCC groups, although NBNC-HCC group was prone to have larger tumor sizes, it did not have more advanced tumors. Meanwhile, there were no significant differences in both 5-year and 10-year disease-free survival and overall survival between NBNC-HCC group and B-HCC or C-HCC group.

**Conclusions:**

Our meta-analysis revealed patients with NBNC-HCC had as worse prognosis as those with hepatitis virus-related HCC. More attention should be paid on patients with non-alcoholic steatohepatitis or metabolic syndromes to prevent the incidence of NBNC-HCC.

**Supplementary Information:**

The online version contains supplementary material available at 10.1186/s12876-023-02925-x.

## Introduction

Hepatocellular carcinoma (HCC), arising from hepatocytes comprising the liver parenchyma, is the sixth most common tumor and the third leading cause of cancer mortality [1]. Among the risk factors of HCC, chronic infection of hepatitis B virus (HBV) or hepatitis C virus (HCV) was the most prominent etiological factor in the development of HCC [2]. However, the HBV vaccination of newborns decreases the incidence of HBV-related HCC (B-HCC) in most countries [3]. Meanwhile, the improvement of blood transfusion techniques, as well as the advent of interferon therapy and new direct-acting antivirals, decreases the incidence of HCV-related HCC (C-HCC) [4, 5]. Unfortunately, the incidence of HBV surface antigen (HBsAg)-negative and HCV antibody (HCVAb)-negative HCC (NBNC-HCC), associated with diabetes mellitus, alcohol-related liver disease (ALD), or non-alcoholic steatohepatitis (NASH), has been reported to be increasing [2, 6].

In the past few years, the comparison of clinical features and prognosis between B-HCC patients and C-HCC patients has been conducted in many studies [7, 8]. Although there were also plenty of researches on NBNC-HCC, the results were not consistent across all publications because of their limited number of patients or differences in demographics [9–11]. In a previous meta-analysis, patients with seronegative virus were considered to have a better prognosis compared to patients with seropositive virus [12]. But some new studies had inconsistent results with the meta-analysis, making the conclusions remain controversial [13–15].

The different etiological factors of HCC can lead to different clinicopathologic characteristics and survival outcomes. The investigation of NBNC-HCC may lead to new insight for the prevention and treatment of HCC. Therefore, the present meta-analysis was performed to update and evaluate the differences in the demographics, oncological features and survival outcomes between NBNC-HCC and hepatitis virus-related HCC.

## Method

### Study selection

A systematic literatures search was performed in PubMed, Embase, Cochrane Library and Web of Science to identify all studies comparing NBNC-HCC with B or/and C-HCC till 2020. The systematic review was conducted according to the Preferred Reporting Items for Systematic reviews and Meta-Analysis (PRISMA) guidelines [16]. The following search headings were used: “hepatitis B virus surface antigen-negative and hepatitis C virus antibody-negative hepatocellular carcinoma”, “non-B non-C hepatocellular carcinoma”, “hepatitis B virus”, “hepatitis C virus”, “hepatocellular carcinoma”, “liver resection” and “hepatectomy”. The initial literature screening was performed by two independent reviewers (Yu, Zhi), who also manually searched the reference lists of the eligible articles for additional studies. Disagreements were resolved after discussion among all authors. The systematic literatures search blinded for the name of authors, institutes and journals to reduce the researcher bias.

### Inclusion and exclusion criteria

HCC were classified into four groups based on the infection status of hepatitis virus: NBNC-HCC group (both HBV and HCV- negative HCC), B-HCC group (HBV-positive and HCV-negative HCC), C-HCC group (HBV-negative and HCV-positive HCC) and BC-HCC group (both HBC and HCV-positive HCC). Included studies had to compare the demographics, clinicopathologic characteristics and survival outcomes of NBNC-HCC group with other groups. If the study populations were reported in more than one publication, the most recent studies with the most complete data were deemed eligible.

The following types of studies were not considered for inclusion in our meta-analysis: 1) studies in which HBV-related and HCV-related HCC patients were accounted into one group, for example, the virus-related HCC group; 2) studies including patients with unresectable lesions or palliative treatments; 3) animal studies, review, case report or cases series, letter to editor and studies not written in English.

### Quality assessment and data extraction

The Newcastle–Ottawa Scale (NOS) was used to assess the quality of each selected study [17]. In the NOS, eight items were categorized into three groups: selection, comparability and outcomes. Each item of the selection and the outcome groups can be allowed for a maximum of one asterisk, while the item of the comparability group can be awarded a maximum of two asterisks. The total score ranged from 0 to 9 asterisks. Studies with five or less asterisks were considered to be low-quality studies and were excluded.

Data was extracted by two independent authors (Yu, Chen) and registered in a spreadsheet for analysis. The extracted information included: general characteristics of studies (authors, year of publication, country, number of patients), patient demographics (age, sex, body mass index (BMI), diabetes mellitus, Child–Pugh grade A), laboratory findings (liver function markers, tumor markers), operative methods (anatomic resection or non-anatomic resection), tumor characteristics (tumor size, tumor number, well-formed capsule, Edmondson-Steiner grade, vascular invasion, liver cirrhosis) and survival data (overall survival (OS), disease-free survival (DFS)). Discrepancies regarding data extraction were solved after discussion among all authors.

### Statistical analysis and evaluation of bias

In the meta-analysis, the extracted data was analyzed using the Review Manager (RevMan) Version 5.4. For dichotomous variables, risk ratio (RR) was used to assess the data, while continuous variables and survival were analyzed using mean difference (MD) and hazard ratio (HR), respectively. When continuous variables were reported using median and range, the mean values and standard deviations were calculated according to the equations proposed by Wan et al*.* [18] and Luo et al*.* [19]. Furthermore, the equations proposed by Tierney et al*.* [20] were used to calculate log HR and its standard error (SE) for survival analysis. We used Mantel–Haenszel method for dichotomous variables, inverse variance method for continuous variables, and the generic inverse variance method for survival analysis.

Statistical heterogeneity was investigated by *Q* test and *I*^2^ statistics. When* P* value of *Q* test was less than 0.1, heterogeneity was considered statistically significant. Furthermore, the *I*^2^ value of 0–24%, 25–49% and 50–100% were interpreted as low, moderate and high heterogeneity, respectively. The publication bias was identified by the visual analysis of the funnel plots. The random effects model was used in data analysis when *I*^2^ value > 50%, while the fixed effects model was used when *I*^2^ value ≤ 50%. *P* < 0.05 was considered statistically significant, and the 95% confidence intervals (CI) were reported in all results.

## Results

### Study characteristics

**Fig. 1:**
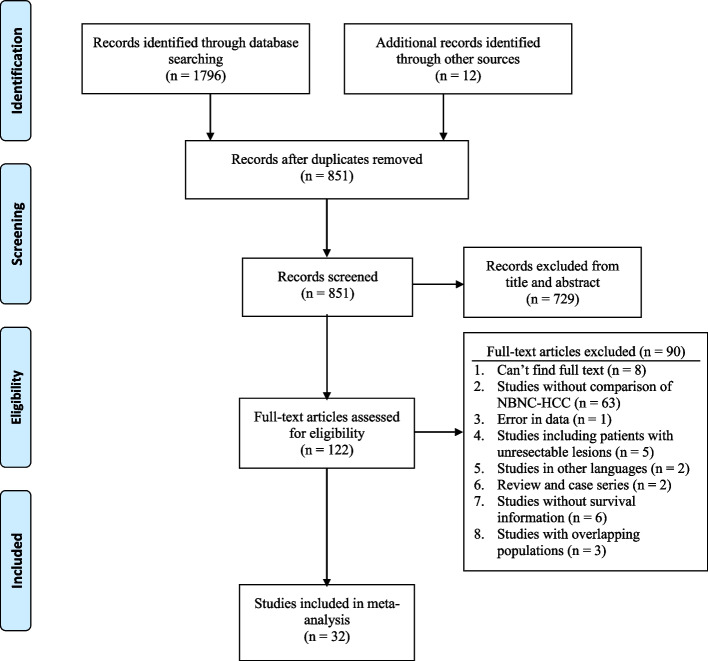
PRISMA flow diagram of the search strategy for studies included in this meta-analysis

The study selection was carried out in accordance with PRISMA flowchart as shown in Fig. 1. Thirty-two studies about NBNC-HCC and hepatitis virus-related HCC were considered eligible, incorporating a total of 26,297 patients with HCC (NBNC-HCC: *n* = 5390, 20.5%; B-HCC: *n* = 9873, 37.5%; C-HCC: *n* = 10,848, 41.3%; BC-HCC: *n* = 186, 0.7%) [9–11, 13–15, 21–46]. All of the studies included in our meta-analysis were comparative cohort studies. More than 90% of the included studies were from Asia, with 19 studies from Japan, 7 studies from China, 2 studies from China (Taiwan) and 1 study from Korea. Besides, there were one study from Italy, one from United States and one from collaborative study among America, France, Japan and China (Hong Kong). None of the studies were excluded because of low NOS score. The characteristics and NOS scores of included studies were summarized in Supplementary Table 1 and Supplementary Table 2.

### Patient demographics

Table 1 summarized the comparation results of the patient demographics among four groups. Patients in NBNC group were significantly older than those in B-HCC group (MD: 10.04 years, *P* < 0.00001) and BC-HCC group (MD: 6.83 years, *P* = 0.04), but similar to those in C-HCC group (MD: 0.19 years, *P* = 0.78). There were more male patients in NBNC-HCC group than in C-HCC group (81.4% vs. 73.7%, *P* < 0.00001). Significant difference in proportion of male was not observed between NBNC-HCC group and B-HCC group (80.4% vs. 84.8%, *P* = 0.25) or BC-HCC group (75.4% vs. 78.0%, *P* = 0.15). The patients with NBNC-HCC had a higher BMI than those with B-HCC (MD: 0.63 kg/m^2^, *P* = 0.01) or C-HCC (MD: 0.82 kg/m^2^, *P* = 0.05). Diabetes mellitus occurred more frequently in NBNC-HCC group compared to B-HCC (40.5% vs. 13.6%, *P* < 0.00001) and C-HCC groups (42.0% vs. 26.0%, *P* < 0.00001). Furthermore, more patients were allocated to Child–Pugh grade A in NBNC-HCC group than other three groups (NBNC-HCC vs. B-HCC: 90.3% vs. 86.6%; NBNC-HCC vs. C-HCC: 89.6% vs. 85.3%; NBNC-HCC vs. BC-HCC: 81.4% vs. 73.5%), with insignificant difference between NBNC-HCC group and B-HCC group (*P* = 0.48).

**Table 1:** Results of patient demographics

Variables	No. of studies	No. of patients	RR/MD	95% CI^a^	Test for overall effect	Heterogeneity	
					*P*	*P*	I^2^ (%)
Age (years)							
NBNC-HCC versus B-HCC	23 [9–11, 14, 15, 21–25, 28–30, 32, 33, 35–40, 43, 46]	7830	10.04	8.55, 11.53	< 0.00001	< 0.00001	92
NBNC-HCC versus C-HCC	21 [9–11, 14, 15, 21–25, 28–30, 32, 33, 35–37, 39, 40, 43]	14,317	0.19	-1.12, 1.50	0.78	< 0.00001	93
NBNC-HCC versus BC-HCC	6 [9, 11, 23, 24, 29, 30]	316	6.83	0.18, 13.48	0.04	< 0.00001	91
Male							
NBNC-HCC versus B-HCC	30 [9–11, 13–15, 21, 23–30, 32–46]	15,160	0.97	0.93, 1.02	0.25	< 0.00001	69
NBNC-HCC versus C-HCC	23 [9–11, 14, 15, 21, 23–30, 32, 33, 35–37, 39, 40, 43, 44]	14,766	1.11	1.06, 1.16	< 0.00001	0.005	49
NBNC-HCC versus BC-HCC	7 [9, 11, 23, 24, 27, 29, 30]	520	0.94	0.86, 1.02	0.15	0.87	0
BMI (kg/m^2^)							
NBNC-HCC versus B-HCC	6 [14, 33, 36, 39, 40, 45]	1183	0.63	0.15, 1.12	0.01	0.04	57
NBNC-HCC versus C-HCC	5 [14, 33, 36, 39, 40]	2211	0.82	0.04, 1.59	0.05	0.33	12
Diabetes mellitus							
NBNC-HCC versus B-HCC	10 [14, 24, 33, 36, 37, 39, 40, 43–45]	1676	2.50	1.87, 3.35	< 0.00001	0.04	49
NBNC-HCC versus C-HCC	9 [14, 24, 33, 36, 37, 39, 40, 43, 44]	2791	1.66	1.36, 2.01	< 0.00001	0.02	57
Child–Pugh grade A							
NBNC-HCC versus B-HCC	19 [11, 13, 15, 22, 24, 26–30, 33, 36–38, 40–42, 44, 46]	13,282	1.02	0.96, 1.08	0.48	< 0.00001	94
NBNC-HCC versus C-HCC	14 [11, 15, 22, 24, 26–30, 33, 36, 37, 40, 44]	12,342	1.04	1.01, 1.06	0.004	0.13	31
NBNC-HCC versus BC-HCC	5 [11, 24, 27, 29, 30]	436	1.11	1.00, 1.22	0.04	0.67	0

*RR* Risk Ratio, *MD* Mean difference, *CI* Confidence Interval, *NBNC-HCC* Both HBV and HCV- negative HCC, *B-HCC* HBV-positive and HCV-negative HCC, *C-HCC* HBV-negative and HCV-positive HCC, *BC-HCC* Both HBC and HCV-positive HCC, *BMI*Body Mass Index

^a^RR was used to assess dichotomous variables (Male, Diabetes mellitus and Child–Pugh grade A), and MD was used to assess continuous variables (Ages and BMI). The NBNC-HCC group was the reference group

### Laboratory findings and operative methods

In terms of liver function, alanine aminotransferase (ALT, MD: -9.62 U/L, *P* < 0.0001), aspartate aminotransferase (AST, MD: -6.57 U/L, *P* = 0.03) and total bilirubin (T-Bil, MD: -0.09 mg/dL, *P* = 0.02) were lower in NBNC-HCC group compared to B-HCC group. ALT (MD: -25.04 U/L, *P* < 0.00001) and AST (MD: -18.39 U/L, *P* < 0.00001) in NBNC-HCC group were also lower than those of the C-HCC group, but the differences in T-Bil between two groups were not significant (MD: -0.07 mg/dL, *P* = 0.09). As for serum albumin and platelet count, the levels of NBNB-HCC group were higher than those of C-HCC group (albumin: MD: 0.23 g/dL, *P* < 0.00001; platelet: MD: 4.66 × 10^4^/μL, *P* < 0.00001). The platelet count in NBNC-group was also higher compared to B-HCC group (MD: 3.21 × 10^4^/μL, *P* < 0.00001), while the difference of albumin was modest between two groups (MD: 0.05 g/dL, *P* = 0.05). There was no significant difference in albumin between NBNC-HCC group and BC-HCC group (MD: 0.23 g/dL, *P* = 0.18). Patients with NBNC-HCC had a lower indocyanine green retention rate at 15 min (ICG-R_15_) than those with C-HCC (MD: -4.85%, *P* < 0.00001), while the difference between NBNC-HCC group and B-HCC group (MD: 0.34%, *P* = 0.33) or BC-HCC group (MD: -0.27%, *P* = 0.93) was not significant.

Regarding tumor markers, alfa-fetoprotein (AFP) level was significantly lower in NBNC-HCC group compared to B-HCC group (MD: -2172.21 ng/mL, *P* = 0.03). Although NBNC-HCC group had a higher AFP level than C-HCC group, the significance was modest (MD: 519.17 ng/mL, *P* = 0.05). No significant difference in AFP level was observed between NBNC-HCC group and BC-HCC group (MD: 558.99 ng/mL, *P* = 0.28). Furthermore, there was no significant difference in des-gamma-carboxy prothrombin (DCP) level between NBNC-HCC group and B-HCC group (MD: 247.02 mAU/mL, *P* = 0.74), while compared to C-HCC group, DCP level was higher in NBNC-HCC group (MD: 1772.52 mAU/mL, *P* = 0.04).

More patients with NBNC-HCC were performed anatomic resection than those with B-HCC (67.1% vs. 55.5%, *P* = 0.03) and those with C-HCC (67.5% vs. 53.4%, *P* = 0.003). However, there was no significant difference in the percentage of anatomic resection between NBNC-HCC group and BC-HCC group (66.8% vs. 72.1%, *P* = 0.76; *I*^2^:0, *P* = 0.63). Table 2 listed the comparison results of the laboratory finding and the operative methods.

**Table 2:** Results of laboratory findings and operative methods

Variables	No. of studies	No. of patients	RR/MD^a^	95% CI	Test for overall effect	Heterogeneity	
					*P*	*P*	I^2^ (%)
ALT (U/L)							
NBNC-HCC versus B-HCC	14 [9, 11, 14, 22, 24, 25, 29, 32, 33, 38, 39, 43, 45]	2126	-9.62	-14.08, -5.17	< 0.0001	< 0.0001	68
NBNC-HCC versus C-HCC	12 [9, 11, 14, 22, 24, 25, 29, 32, 33, 39, 40, 43]	3100	-25.04	-34.95, -15.13	< 0.00001	< 0.00001	93
NBNC-HCC versus BC-HCC	4 [9, 11, 24, 29]	213	-29.34	-43.25, -15.43	< 0.0001	0.39	1
AST (U/L)							
NBNC-HCC versus B-HCC	13 [9, 11, 14, 22, 24, 25, 28, 32, 35, 38–40, 43]	1672	-6.57	-12.59, -0.55	0.03	< 0.0001	71
NBNC-HCC versus C-HCC	11 [9, 11, 14, 22, 24, 25, 32, 35, 39, 40, 43]	3003	-18.39	-24.47, -12.32	< 0.00001	< 0.00001	82
NBNC-HCC versus BC-HCC	3 [9, 11, 24]	126	-34.91	-74.48, 4.67	0.08	0.0005	87
T-Bil (mg/dL)							
NBNC-HCC versus B-HCC	15 [11, 14, 15, 21, 24, 28, 29, 32, 33, 35, 36, 38–40, 43]	7088	-0.09	-0.16, -0.01	0.02	< 0.00001	84
NBNC-HCC versus C-HCC	12 [11, 14, 21, 24, 29, 32, 33, 35, 36, 39, 40, 43]	3472	-0.07	-0.16, -0.01	0.09	< 0.00001	84
NBNC-HCC versus BC-HCC	3 [11, 24, 29]	189	-0.48	-1.27, 0.32	0.24	< 0.00001	93
Albumin (g/dL)							
NBNC-HCC versus B-HCC	17 [9, 11, 14, 15, 21, 25, 28, 29, 33, 35–40, 42, 43]	8439	0.05	-0.00, 0.09	0.05	< 0.00001	89
NBNC-HCC versus C-HCC	14 [9, 11, 14, 21, 25, 28, 29, 33, 35–37, 39, 40, 43, 44]	3903	0.23	0.15, 0.31	< 0.00001	< 0.00001	81
NBNC-HCC versus BC-HCC	3 [9, 11, 29]	160	0.23	-0.11, 0.56	0.18	0.02	75
Platelet (× 10^4^/μL)							
NBNC-HCC versus B-HCC	12 [11, 14, 25, 29, 32, 33, 35, 36, 38–40, 43]	1961	3.21	2.40, 4.02	< 0.00001	0.37	8
NBNC-HCC versus C-HCC	11 [11, 14, 25, 29, 32, 33, 35, 36, 39, 40, 43]	3478	4.66	3.72, 5.60	< 0.00001	0.04	48
ICG-R_15_ (%)							
NBNC-HCC versus B-HCC	14 [9, 21–25, 32, 33, 35, 36, 39, 40, 42, 43]	3117	0.34	-0.34, 1.02	0.33	< 0.00001	75
NBNC-HCC versus C-HCC	13 [9, 21–25, 32, 33, 35, 36, 39, 40, 43]	3324	-4.85	-6.04, -3.65	< 0.00001	0.0003	67
NBNC-HCC versus BC-HCC	3 [9, 23, 24]	137	-0.27	-5.82, 5.29	0.93	0.09	59
AFP (ng/mL)							
NBNC-HCC versus B-HCC	13 [9, 11, 14, 21, 25, 30, 32, 33, 35, 39, 40, 43, 46]	1741	-2172.21	-4081.13, -263.30	0.03	< 0.00001	83
NBNC-HCC versus C-HCC	12 [9, 11, 14, 21, 25, 30, 32, 33, 35, 39, 40, 43]	3581	519.17	-1.00, 1039.35	0.05	0.33	12
NBNC-HCC versus BC-HCC	3 [9, 11, 30]	116	558.99	-464.56, 1582.54	0.28	0.20	38
DCP (mAU/mL)							
NBNC-HCC versus B-HCC	6 [14, 30, 33, 35, 40, 43]	1002	247.02	-1210.87, 1704.91	0.74	0.20	31
NBNC-HCC versus C-HCC	6 [14, 30, 33, 35, 40, 43]	2102	1772.52	57.20, 3487.85	0.04	0.03	60
Anatomic resection							
NBNC-HCC versus B-HCC	12 [11, 14, 29, 30, 32–36, 40, 41, 44]	2619	1.14	1.01, 1.29	0.03	< 0.0001	72
NBNC-HCC versus C-HCC	10 [11, 14, 29, 30, 32, 33, 35, 36, 40, 44]	2937	1.24	1.08, 1.43	0.003	< 0.0001	79
NBNC-HCC versus BC-HCC	3 [11, 29, 30]	179	0.97	0.82, 1.16	0.76	0.63	0

*RR* Risk Ratio, *MD* Mean difference, *CI* Confidence Interval, *NBNC-HCC* Both HBV and HCV- negative HCC, *B-HCC* HBV-positive and HCV-negative HCC, *C-HCC* HBV-negative and HCV-positive HCC, *BC-HCC* Both HBC and HCV-positive HCC, *ALT* Alanine aminotransferase, *AST* Aspartate aminotransferase, *T-Bil* Total bilirubin, *ICG-R*_*15*_ Indocyanine green retention rate at 15 min AFP, alfa-fetoprotein, *DCP* Des-gamma-carboxy prothrombin

^a^RR was used to assess dichotomous variables (Anatomic resection), and MD was used to assess continuous variables (ALT, AST, T-Bil, Albumin, Platelet, ICG-R_15_, AFP and DCP). The NBNC-HCC group was the reference group

### Tumor characteristics

The results of tumor characteristics were shown in Table 3. Patients with NBNC-HCC group had larger tumor sizes than those with C-HCC group (MD: 1.29 cm, *P* < 0.00001), but no significant difference was observed between NBNC-HCC group and B-HCC group (MD: 0.52 cm, *P* = 0.09) or BC-HCC group (MD: -0.12 cm, *P* = 0.69). In terms of tumor number, patients with NBNC-HCC had less multiple tumors than patients with B-HCC (19.8% vs. 20.4%, *P* = 0.005) and patients with C-HCC (21.7% vs. 26.9%, *P* = 0.004). The percentage of well-formed capsules was similar between NBNC-HCC group and B-HCC group (55.9% vs. 55.8%, *P* = 0.95) or BC-HCC group (45.2% vs. 42.2%, *P* = 0.46), while less tumors formed capsules in NBNC-HCC group than in C-HCC group (62.9% vs. 68.2%, *P* = 0.03). Regarding tumor differentiation, patients with NBNC-HCC had similar Edmondson-Steiner grade I + II tumors to those with B-HCC (64.6% vs. 66.8%, *P* = 0.85) and those with C-HCC (48.3% vs. 41.2%, *P* = 0.38). Furthermore, NBNC-HCC group had less incidence of vascular invasion than B-HCC group (28.2% vs. 32.3%, *P* = 0.008) and BC-HCC group (18.1% vs. 27.9%, *P* = 0.01), while no significant difference in incidence of vascular invasion was observed between NBNC-HCC group and C-HCC group (33.1% vs. 36.6%, *P* = 0.64). The prevalence of liver cirrhosis was significantly lower in NBNC-HCC group than in B-HCC group (32.2% vs. 66.6%, *P* < 0.00001), C-HCC group (28.7% vs. 49.5%, *P* < 0.00001) and BC-HCC group (16.4% vs. 22.8%, *P* < 0.00001).

**Table 3:** Results of tumor characteristics

Variables	No. of studies	No. of patients	RR/MD^a^	95% CI	Test for overall effect	Heterogeneity	
					*P*	*P*	I^2^ (%)
Tumor size (cm)							
NBNC-HCC versus B-HCC	15 [9, 11, 14, 23, 25, 29, 32, 33, 35, 36, 38–40, 43, 45]	2485	0.52	-0.08, 1.11	0.09	< 0.00001	95
NBNC-HCC versus C-HCC	13 [9, 11, 14, 23, 25, 29, 32, 33, 35, 36, 39, 40, 43]	3759	1.29	0.93, 1.66	< 0.00001	< 0.00001	83
NBNC-HCC versus BC-HCC	4 [9, 11, 23, 29]	220	-0.12	-0.70, 0.46	0.69	0.006	76
Multiple tumors							
NBNC-HCC versus B-HCC	24 [9, 10, 13–15, 21, 23, 25–30, 32–34, 36–38, 40, 42–45]	13,814	0.82	0.71, 0.94	0.005	0.04	36
NBNC-HCC versus C-HCC	19 [9, 10, 14, 15, 21, 23, 25–30, 32, 33, 36, 37, 40, 43, 44]	13,494	0.79	0.67, 0.93	0.004	0.04	39
NBNC-HCC versus BC-HCC	5 [9, 23, 27, 29, 30]	418	0.59	0.29, 1.21	0.15	0.07	53
Well-formed capsule							
NBNC-HCC versus B-HCC	14 [10, 11, 13, 14, 23, 24, 29, 34, 36–38, 40, 41]	6745	1.00	0.89, 1.11	0.95	< 0.00001	76
NBNC-HCC versus C-HCC	10 [10, 11, 14, 23, 24, 28, 29, 36, 37, 40]	2109	0.92	0.85, 0.99	0.03	0.28	18
NBNC-HCC versus BC-HCC	4 [11, 23, 24, 29]	249	1.11	0.84, 1.47	0.46	0.63	0
Edmondson-Steiner grade (I + II)							
NBNC-HCC versus B-HCC	6 [11, 13, 22, 23, 34, 38]	5104	1.01	0.93, 1.09	0.85	0.19	33
NBNC-HCC versus C-HCC	3 [11, 22, 23]	308	1.19	0.81, 1.76	0.38	0.12	53
Vascular invasion							
NBNC-HCC versus B-HCC	15 [9, 11, 13, 14, 23, 24, 29, 30, 33, 38, 39, 42, 44–46]	7851	0.79	0.66, 0.94	0.008	0.001	61
NBNC-HCC versus C-HCC	10 [9, 11, 14, 23, 24, 29, 30, 33, 39, 44]	2433	0.95	0.78, 1.16	0.64	0.08	42
NBNC-HCC versus BC-HCC	5 [11, 23, 24, 29, 30]	292	0.60	0.40, 0.89	0.01	0.45	0
Liver cirrhosis							
NBNC-HCC versus B-HCC	24 [9, 10, 13–15, 21–24, 27–30, 32, 33, 35–37, 39, 40, 42, 44–46]	13,837	0.66	0.58, 0.76	< 0.00001	< 0.00001	85
NBNC-HCC versus C-HCC	20 [9, 10, 14, 15, 21–24, 27–30, 32, 33, 35–37, 39, 40, 44]	14,231	0.64	0.56, 0.73	< 0.00001	< 0.00001	71
NBNC-HCC versus BC-HCC	6 [9, 23, 24, 27, 29, 30]	471	0.65	0.53, 0.81	< 0.00001	0.17	36

*RR* Risk Ratio, MD, mean difference, *CI* Confidence Interval, *NBNC-HCC* both HBV and HCV- negative HCC, *B-HCC* HBV-positive and HCV-negative HCC, *C-HCC* HBV-negative and HCV-positive HCC, *BC-HCC* both HBC and HCV-positive HCC

^a^RR was used to assess dichotomous variables (Multiple tumors, Well-formed capsule, Edmondson-Steiner grade (I + II), Vascular invasion and Liver cirrhosis), and MD was used to assess continuous variables (Tumor size). The NBNC-HCC group was the reference group

### Survival analysis

**Fig. 2:**
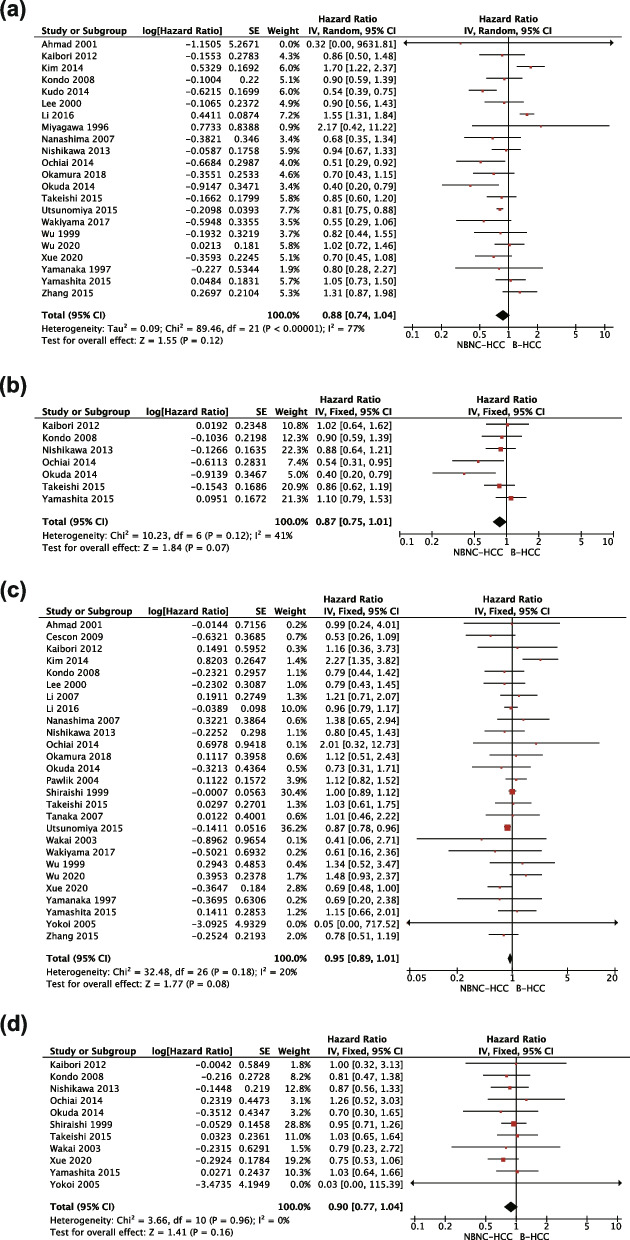
**a** Forest plot for the result from all studies comparing 5-year disease-free survival between NBNC-HCC and B-HCC groups. **b** Forest plot for the result from all studies comparing 10-year disease-free survival between NBNC-HCC and B-HCC groups. **c** Forest plot for the result from all studies comparing 5-year overall survival between NBNC-HCC and B-HCC groups. **d** Forest plot for the result from all studies comparing 10-year overall survival between NBNC-HCC and B-HCC groups

**Fig. 3:**
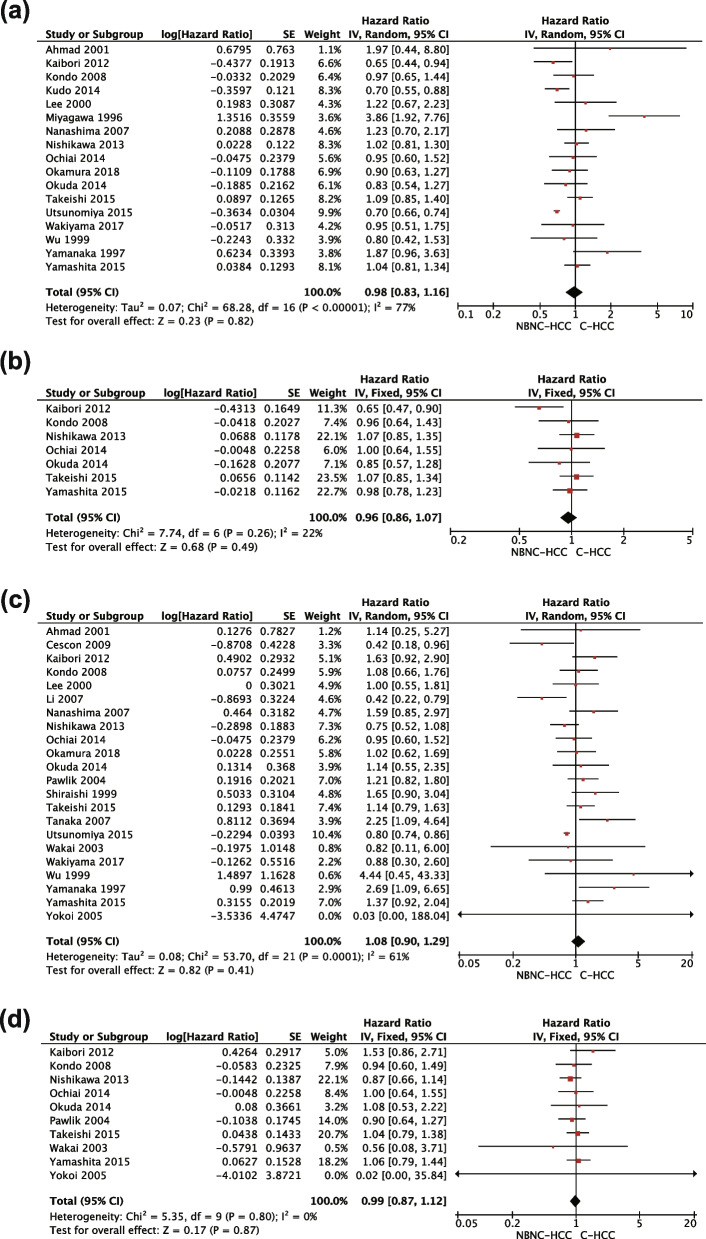
**a** Forest plot for the result from all studies comparing 5-year disease-free survival between NBNC-HCC and C-HCC groups. **b** Forest plot for the result from all studies comparing 10-year disease-free survival between NBNC-HCC and C-HCC groups. **c** Forest plot for the result from all studies comparing 5-year overall survival between NBNC-HCC and C-HCC groups. **d** Forest plot for the result from all studies comparing 10-year overall survival between NBNC-HCC and C-HCC groups

There were no significant differences in 5-year DFS (HR: 0.88, *P* = 0.12, Fig. 2a) and 10-year DFS (HR: 0.84, *P* = 0.10, Fig. 2b), as well as 5-year OS (HR: 0.97, *P* = 0.48, Fig. 2c) and 10-year OS (HR: 0.90, *P* = 0.16, Fig. 2d) between NBNC-HCC group and B-HCC group. In addition, no significant difference was observed between NBNC-HCC group and C-HCC group in 5-year DFS (HR: 0.98, *P* = 0.82, Fig. 3a) and 10- year DFS (HR: 0.95, *P* = 0.46, Fig. 3b), as well as 5-year OS (HR: 1.08, *P* = 0.41, Fig. 3c) and 10-year (HR: 0.99, *P* = 0.87, Fig. 3d). Because of the limited number of patients, the analysis of survival between NBNC-HCC group and BC-HCC group was not conducted.

Subgroup analyses of survival outcomes between NBNC-HCC group and B-HCC group (Supplementary Table 3) or C-HCC group (Supplementary Table 4) were performed based on different regions (China and Japan) and different number of cases (< 100 cases/ > 100 cases). The pooled results of studies conducted in China showed that there was not significant difference in survival outcomes, including 5-year DFS and 5-year OS, between NBNC-HCC group and B-HCC group or C-HCC group. In studies of Japan, the pooled results revealed that patients with NBNC-HCC had a better 5-year DFS (HR: 0.77, *P* < 0.0001) compared to those with B-HCC and a better 5-year OS (HR: 0.87, *P* < 0.0001) compared to those with C-HCC. Subgroup analyses based on different number of cases revealed that no significant difference in survival outcomes was observed between NBNC-HCC group and B-HCC or C-HCC group.

### Publication bias

Although some funnel plots were roughly symmetrical, most of the funnel plots were asymmetrical, suggesting that there were publication biases among the studies (Supplementary Fig. 1).

## Discussion

Different hepatitis virus infection status can lead to different pathogenic mechanisms of hepatocarcinogenesis, thus affecting the clinicopathologic characteristics and prognosis of HCC [6, 34–36, 47]. In this circumstance, our meta-analysis including 32 eligible studies was conducted to compare the clinical characteristics and survival outcomes between NBNC-HCC and hepatitis viruses positive HCC, aiming to draw some clinically meaningful conclusions.

It is generally assumed that the vertical transmission of HBV in infancy leads to occurrence of HCC at a younger age in the most patients with HBV infection, while NBNC-HCC usually manifests later in life [12, 25, 34, 41]. In accordance with this notion, our meta-analysis showed the average age at the occurrence of HCC was higher in NBNC-HCC than in B-HCC and BC-HCC. On the other hand, different from HBV infection, HCV infection usually occurs after the age of 20 years [12]. Regarding the comparison of the average age between NBNC-HCC and C-HCC, the results differed among the studies, but no significant difference was observed after the results were analyzed. In addition, HCC is more common in males than in females [48, 49]. The previous study supposed that the lower percentage of female patients with NBNC-HCC resulted from the condition that most patients with habitual alcohol consumption were male [15]. In terms of the sex differences among the four groups, the proportion of male patients in C-HCC group were lower than that in NBNC-HCC group. However, the reasons of the differences needed to be investigated furtherly.

More and more studies demonstrated nonalcoholic steatohepatitis (NASH) and metabolic syndrome, including diabetes mellitus and obesity, were significant risk factors in the development of NBNC-HCC [2, 40, 43]. In a previous study, the BMI ≥ 23 kg/m^2^ was even regarded as an important factor which influenced DFS of HCC patients [14]. In present analysis, although several included studies reported different results, the overall result showed that BMI and the percentage of diabetes mellitus were higher in the NBNC-HCC group.

Regarding liver function, the results of difference in proportion of Child–Pugh grade A, as well as the analysis of laboratory findings and liver cirrhosis, showed that the liver function in NBNC-HCC group was better compared to B-HCC and C-HCC groups. This result may be explained by the aspect of different oncological characteristics in HCC. In patients with NBNC-HCC, the conventional multistep progress, defined as fibrosis-cirrhosis-hepatocarcinogenesis, was not the main pathophysiological mechanism, and the tumors were characterized by the lower carcinogenic potential, fewer host DNA mutations and less chronic necro-inflammatory reaction, which resulted in the better liver function [14, 40, 45]. However, for patients with HCV infection, the existence of virus induced persistent inflammation, fibrosis and subsequent cirrhosis, thus leading to HCC eventually [27, 29, 50]. In general, the severe inflammation led to the poor liver function in patients with C-HCC. Therefore, our meta-analysis showed that the liver function of patients in C-HCC group was the worse than those in NBNC-HCC group.

For patients with NBNC-HCC, owing to the less regular medical surveillance and the fewer symptoms compared to hepatitis virus-related HCC, the tumors were detected at an advanced stage with a larger tumor size [14, 33, 34]. Consequently, our meta-analysis showed that tumors of NBNC-HCC were larger than those of C-HCC. Since the better liver function reserve and larger tumor sizes may necessitate major resection to achieve an R0 margin, more patients with NBNC-HCC were performed anatomic resection, which was proposed to be superior to non-anatomic resection in removing the entire tumor burden and potential metastatic lymph nodes [33, 38, 51]. Furthermore, it was considered in some studies that capsule formation was the characteristic of HCC developing in the setting of liver cirrhosis and was also the part of the defense mechanisms against the growth of HCC [13, 52]. Therefore, in our meta-analysis, the setting of severe liver cirrhosis of C-HCC led to more formation of capsules, resulting in the smaller tumor size furtherly. However, there was no significant difference in tumor size between NBNC-HCC and B-HCC groups. In other tumor characteristics, the proportion of multiple tumors was the lowest in NBNC-group. The percentage of Edmonson-Steiner grade I + II tumors in NBNC-HCC group was not significant different from that of B-HCC and C-HCC groups, while the incidence of vascular invasion was lower compared to B-HCC group. To sum up, the pooled results of tumor characteristics in our analysis demonstrated that the tumors in NBNC-HCC group were not more advanced than other groups.

HCC recurrence after surgery was one of the most serious problems in the treatment for HCC [25]. There were two types of HCC recurrence, including “early recurrence” mainly because of intrahepatic metastasis (IM) and “late recurrence” because of multicentric (MC) hepatocarcinogenesis [53]. It was proposed that compared to B-HCC, NBNC-HCC has the lower risk of IM recurrence, while the risk of MC recurrence of NBNC-HCC was lower than that of C-HCC, and patients with NBNC-HCC were proved to have a better prognosis than those with B-HCC or C-HCC [15]. However, our meta-analysis drew a different conclusion and showed that patients with NBNC-HCC had comparable prognosis to those with B-HCC or C-HCC, which was also inconsistent with the results of previous meta-analysis [12]. In our opinion, the prognosis of HCC might be affected by many factors. For example, although more choices of anatomic resection occurred in the NBNC-HCC group, the remnant liver volume might not permit another curative resection when the recurrence occurred [54]. Because of inter-study heterogeneity, we performed the subgroup analyses based on different regions and different number of cases. Except for the pooled results of 5-year DFS between NBNC-HCC and B-HCC groups based on studies from Japan and 5-year OS between NBNC-HCC and C-HCC groups based on studies from Japan, the majority of pooled results confirmed that no significant difference in survival outcomes was observed between NBNC-HCC and hepatitis virus-related HCC. We speculated that this may be related to the different etiology of HCC in Japan from in China.

This meta-analysis had several limitations. First, there was significant inter-study heterogeneity in some analysis of variables, which might influence the final conclusion. Second, the majority of eligible studies were single center researches with limited number of patients, representing a relatively lower level of clinical evidence. Third, most of the included studies were from Asian institutions, and it was demonstrated that the diagnosis and the treatment of HCC could be affected by ethnicity [46]. As such, the conclusion of the present analysis might be more applicable to Asian population. Besides, patients with hepatitis B core antibody (HBcAb) positive but HBsAg-negative, which indicated a history of infection, might be divided into the NBNC-HCC group in the included studies. However, the HBcAb-positive was proposed to be an important risk factor of recurrence and poor prognosis, which might influence the results. Finally, although the comparison between the BC-HCC group and other groups were performed, some results needed to be confirmed by further researches because of the complex mutual competition between HBV and HCV as well as the small sample size in the analysis [29, 30].

To be concluded, patients of NBNC-HCC showed better liver function and lighter liver cirrhosis compared with patients of hepatitis virus-related HCC. Although the tumors in the NBNC-HCC group were not more advanced, the survival outcomes in the NBNC-HCC patients didn’t improve compared with hepatitis virus-related HCC patients. Thus, healthy diet and lifestyle should be formed to prevent the incidence of NASH and metabolic syndrome. Furthermore, medical intervention to primary diseases and regular medical surveillance of liver is necessary for patients with NASH or metabolic syndrome to detect the HCC at the early stage.

### Supplementary Information

**Additional file 1: Supplementary Fig 1. **(a) Funnel plot for the result from all studies comparing 5-year overall survival between NBNC-HCC and B-HCC groups. (b) Funnel plot for the result from all studies comparing 5-year disease-free survival between NBNC-HCC and C-HCC groups.

**Additional file 2: Supplementary Table 1. **General Characteristics of Studies Included in the Meta-analysis. **Supplementary Table 2. **Quality assessment of studies pooled in the meta-analysis based on the Newcastle-Ottawa Scale. **Supplementary Table 3. **Subgroup analyses of survival outcomes between NBNC-HCC and B-HCC groups. **Supplementary Table 4. **Subgroup analyses of survival outcomes between NBNC-HCC and C-HCC groups. 

## Data Availability

Not applicable in our study.
