# Supplementary figures and images for: Comparison of clinicopathologic characteristics among patients with HBV-positive, HCV-positive and Non-B Non-C hepatocellular carcinoma after hepatectomy: a systematic review and meta-analysis
Source: BMC Gastroenterol. 2023 Aug 23;23:289. doi: 10.1186/s12876-023-02925-x (PMC10463328; doi:10.1186/s12876-023-02925-x)

**(a)**

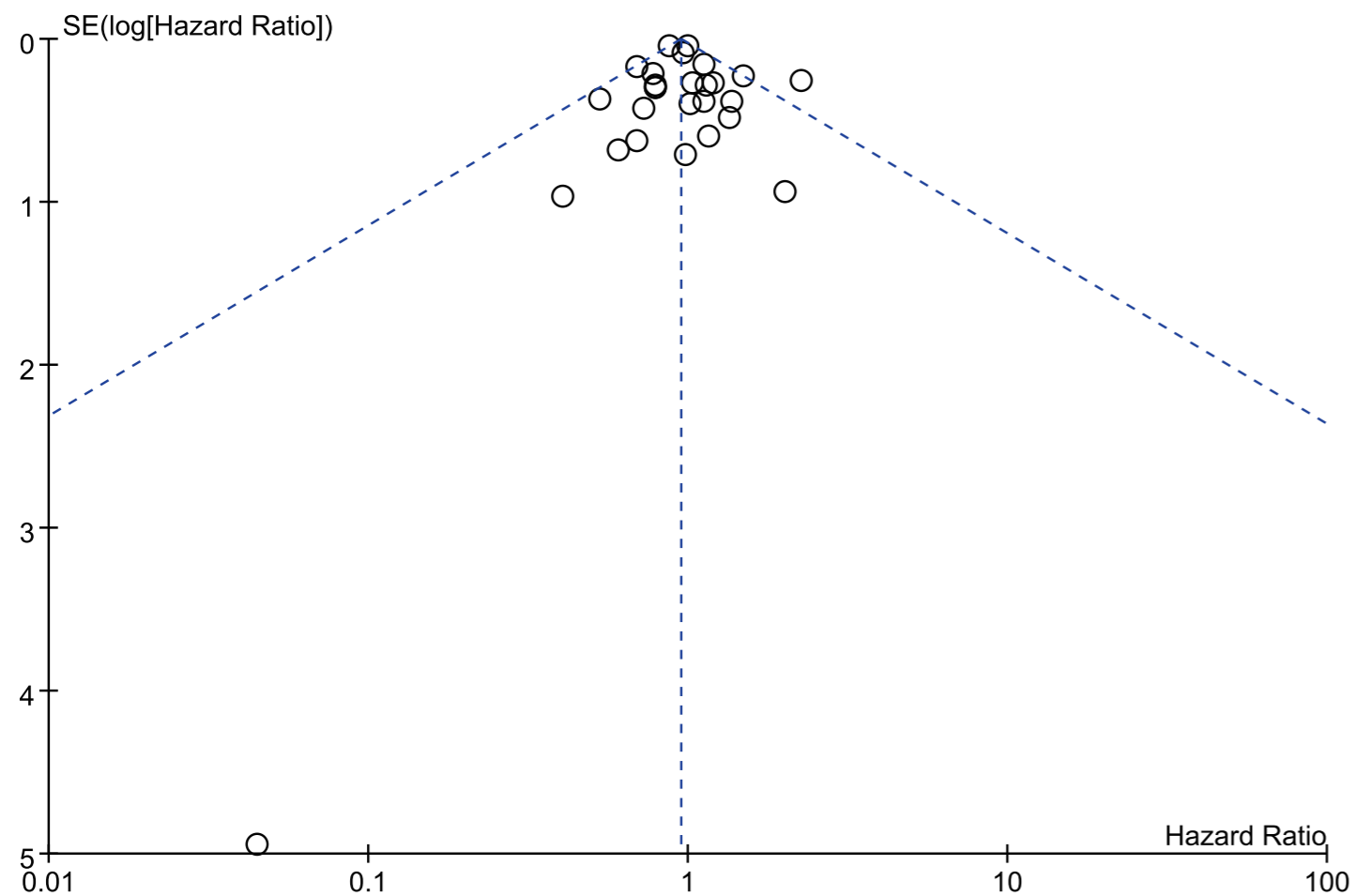

**(b)**

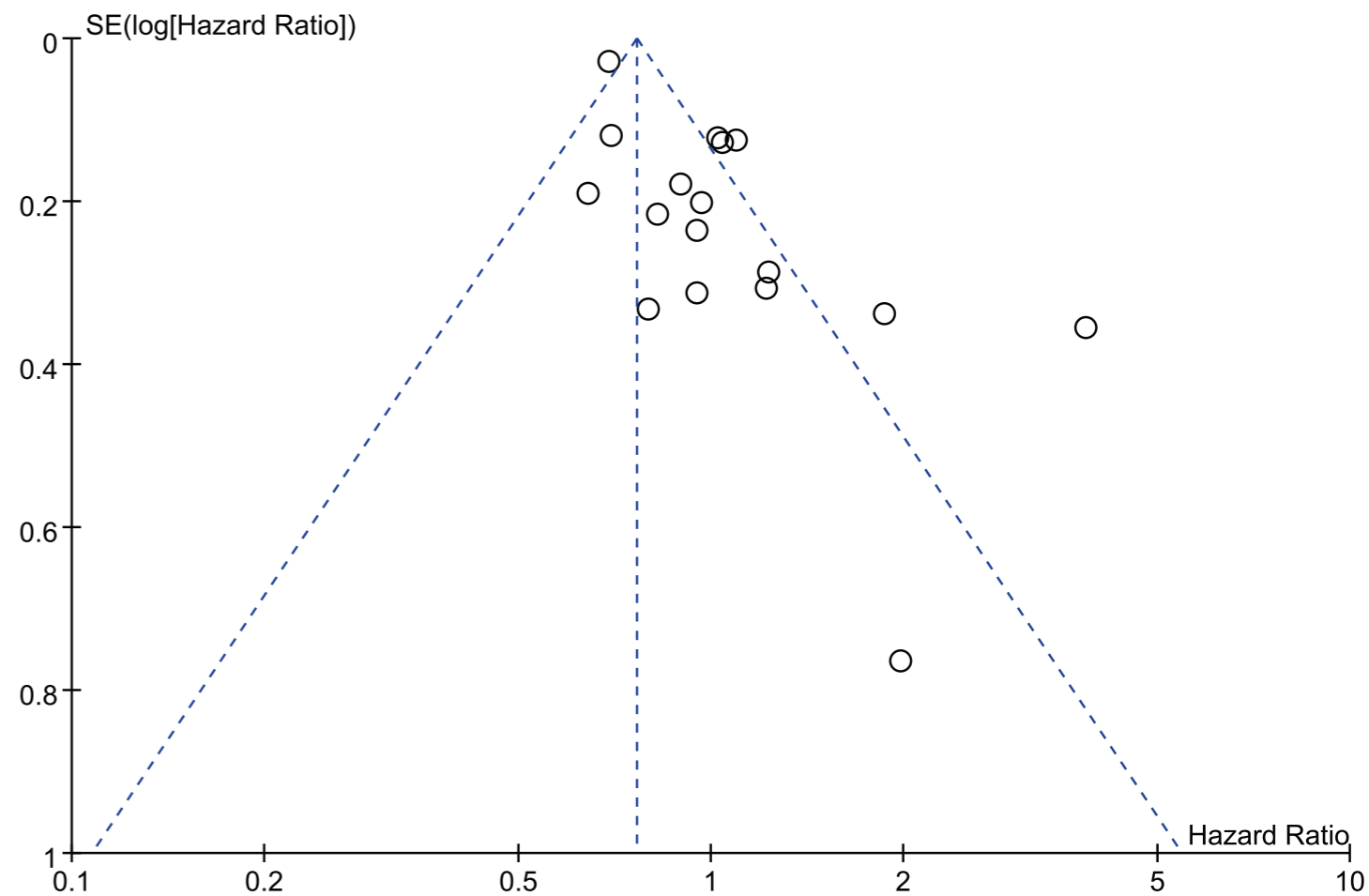

Supplement: Supplementary file 1 — Additional file 1: Supplementary Fig 1. (a) Funnel plot for the result from all studies comparing 5-year overall survival between NBNC-HCC and B-HCC groups. (b) Funnel plot for the result from all studies comparing 5-year disease-free survival between NBNC-HCC and C-HCC groups. [file 12876_2023_2925_MOESM1_ESM.pdf]
