# Supplementary material for: Comparison of clinicopathologic characteristics among patients with HBV-positive, HCV-positive and Non-B Non-C hepatocellular carcinoma after hepatectomy: a systematic review and meta-analysis
Source: BMC Gastroenterol. 2023 Aug 23;23:289. doi: 10.1186/s12876-023-02925-x (PMC10463328; doi:10.1186/s12876-023-02925-x)
Supplement: Supplementary file 2 — Additional file 2: Supplementary Table 1. General Characteristics of Studies Included in the Meta-analysis. Supplementary Table 2. Quality assessment of studies pooled in the meta-analysis based on the Newcastle-Ottawa Scale. Supplementary Table 3. Subgroup analyses of survival outcomes between NBNC-HCC and B-HCC groups. Supplementary Table 4. Subgroup analyses of survival outcomes between NBNC-HCC and C-HCC groups. [file 12876_2023_2925_MOESM2_ESM.docx]

**Supplementary Table 1 General Characteristics of Studies Included in the Meta-analysis**

| Author | Year | Country | Inclusion Period | No. of patients | No. of patients in different groups (%) | | | | Sex (Male/Female) | | | |
| --- | --- | --- | --- | --- | --- | --- | --- | --- | --- | --- | --- | --- |
|  |  |  |  |  | B-HCC | C-HCC | NBNC-BCC | BC-HCC | B-HCC | C-HCC | NBNC-HCC | BC-HCC |
| Miyagawa [21] | 1996 | Japan | 1990-1995 | 175 | 32 | 124 | 19 | - | 21/11 | 96/28 | 15/4 | - |
| Yamanaka [9] | 1997 | Japan | 1991-1994 | 202 | 27 | 151 | 20 | 4 | 24/3 | 125/26 | 18/2 | 3/1 |
| Wu [23] | 1999 | China (Taiwan) | 1990-1997 | 261 | 131 | 70 | 40 | 20 | 110/21 | 56/14 | 29/11 | 16/4 |
| Shiraishi [22] | 1999 | Japan | 1991-1996 | 44 | 11 | 21 | 12 | - | - | - | - | - |
| Lee [24] | 2000 | China (Taiwan) | 1992-1998 | 252 | 133 | 66 | 30 | 23 | 112/21 | 48/18 | 20/10 | 16/7 |
| Noguchi [25] | 2000 | Japan | 1991-1998 | 289 | 44 | 232 | 13 | - | 34/10 | 172/60 | 12/1 | - |
| Ahmad [26] | 2001 | U. S | 1990-1998 | 77 | 17 | 44 | 16 | - | 12/5 | 34/10 | 6/10 | - |
| Wakai [10] | 2003 | Japan | 1990-1999 | 111 | 32 | 55 | 24 | - | 20/12 | 46/9 | 18 | - |
| Pawlik [27] | 2004 | Multi centers | 1990-2000 | 446 | 163 | 79 | 126 | 78 | 137/26 | 48/31 | 90/36 | 57/21 |
| Yokoi [28] | 2005 | Japan | 1990-2000 | 154 | 25 | 116 | 13 | - | 19/6 | 95/21 | 10/3 | - |
| Li [29] | 2007 | China | 1996-2003 | 413 | 251 | 75 | 54 | 33 | 212/39 | 62/13 | 44/10 | 27/6 |
| Nanashima [30] | 2007 | Japan | 1990-2005 | 243 | 76 | 124 | 29 | 14 | 61/15 | 99/25 | 21/8 | 12/2 |
| Tanaka [31] | 2007 | Japan | 1992-2005 | 210 | 46 | 130 | 34 | - | - | - | - | - |
| Kondo [32] | 2008 | Japan | 1990-2006 | 265 | 78 | 127 | 60 | - | 58/20 | 94/33 | 43/17 | - |
| Cescon [11] | 2009 | Italy | 1997-2006 | 204 | 25 | 130 | 35 | 14 | 24/1 | 90/40 | 30/5 | 14/0 |
| Kaibori [33] | 2012 | Japan | 1992-2009 | 496 | 85 | 351 | 60 | - | 68/17 | 272/79 | 52/8 | - |
| Li [13] | 2013 | China | 1997-2009 | 4204 | 3529 | - | 675 | - | 3070/459 | - | 521/154 | - |
| Nishikawa [14] | 2013 | Japan | 1999-2012 | 475 | 62 | 284 | 129 | - | 42/20 | 194/90 | 104/25 | - |
| Kim [34] | 2014 | Korea | 2005-2010 | 463 | 360 | - | 103 | - | 290/70 | - | 85/18 | - |
| Ochiai [36] | 2014 | Japan | 1990-2011 | 284 | 49 | 174 | 61 | - | 40/9 | 112/62 | 56/5 | - |
| Okuda [37] | 2014 | Japan | 2000-2013 | 201 | 32 | 93 | 76 | - | 23/9 | 70/23 | 67/9 | - |
| Kudo [35] | 2014 | Japan | 2000-2012 | 539 | 96 | 275 | 168 | - | 74/22 | 200/75 | 141/27 | - |
| Zhou [38] | 2014 | China | 2006-2009 | 183 | 124 | - | 59 | - | 106/18 | - | 42/17 | - |
| Takeishi [39] | 2015 | Japan | 1989-2013 | 662 | 94 | 451 | 117 | - | 71/23 | 290/261 | 88/29 | - |
| Utsunomiya [15] | 2015 | Japan | 2000-2005 | 11950 | 2194 | 7018 | 2738 | - | 1796/398 | 5225/1793 | 2253/485 | - |
| Yamashita [40] | 2015 | Japan | 1990-2011 | 694 | 110 | 474 | 110 | - | 83/27 | 304/170 | 81/29 | - |
| Zhang [41] | 2015 | China | 2006-2014 | 473 | 409 | - | 64 | - | 373/36 | - | 53/11 | - |
| Li [42] | 2016 | China | 2008-2012 | 1440 | 1200 | - | 240 | - | 1070/130 | - | 172/68 | - |
| Wakiyama [43] | 2017 | Japan | 2000-2010 | 134 | 36 | 57 | 4 | - | 33/3 | 46/11 | 36/5 | - |
| Okamura [44] | 2018 | Japan | 2002-2010 | 272 | 55 | 127 | 90 | - | 37/17 | 104/23 | 79/11 | - |
| Wu [45] | 2020 | China | 2010-2012 | 306 | 240 | - | 66 | - | 208/32 | - | 53/13 | - |
| Xue [46] | 2020 | China | - | 175 | 107 | - | 68 | - | 98/9 | - | 58/10 | - |
| Total |  |  |  | 26297 | 9873 (37.5%) | 10848 (41.3%) | 5390 (20.5%) | 186 (0.7%) | 8326/1489 | 7882/2915 | 4297/1041 | 145/41 |

NOS, Newcastle-Ottawa Scale; NBNC-HCC, both HBV and HCV- negative HCC; B-HCC, HBV-positive and HCV-negative HCC; C-HCC, HBV-negative and HCV-positive HCC; BC-HCC, both HBC and HCV-positive HCC

**Supplementary Table 2 Quality assessment of studies pooled in the meta-analysis based on the Newcastle-Ottawa Scale**

| Author | Selection ^†^ | Comparability ^‡^ | Outcome^§^ | Quality judgement |
| --- | --- | --- | --- | --- |
| Miyagawa [21] | *** | * | *** | 7 |
| Yamanaka [9] | *** |  | *** | 6 |
| Wu [23] | *** | * | *** | 7 |
| Shiraishi [22] | *** | * | *** | 7 |
| Lee [24] | *** | * | *** | 7 |
| Noguchi [25] | *** | ** | ** | 7 |
| Ahmad [26] | *** | ** | *** | 8 |
| Wakai [10] | *** | * | *** | 7 |
| Pawlik [27] | *** |  | *** | 6 |
| Yokoi [28] | *** | * | *** | 7 |
| Li [29] | *** | * | *** | 7 |
| Nanashima [30] | *** | * | *** | 7 |
| Tanaka [31] | *** | * | *** | 7 |
| Kondo [32] | *** | * | *** | 7 |
| Cescon [11] | *** |  | *** | 6 |
| Kaibori [33] | *** | * | *** | 7 |
| Li [13] | *** |  | *** | 6 |
| Nishikawa [14] | *** |  | *** | 6 |
| Kim [34] | *** | * | *** | 7 |
| Ochiai [36] | *** |  | *** | 6 |
| Okuda [37] | *** | * | *** | 7 |
| Kudo [35] | *** |  | *** | 6 |
| Zhou [38] | *** |  | *** | 6 |
| Takeishi [39] | *** | * | *** | 7 |
| Utsunomiya [15] | *** |  | *** | 6 |
| Yamashita [40] | *** | * | *** | 7 |
| Zhang [41] | *** |  | *** | 6 |
| Li [42] | *** |  | *** | 6 |
| Wakiyama [43] | *** | * | *** | 7 |
| Okamura [44] | *** |  | *** | 6 |
| Wu [45] | *** | * | ** | 6 |
| Xue [46] | *** | * | *** | 7 |

^†^Selection: (1) representativeness of the exposed cohort: (a) truly representative of the average patients with NBNC-HCC in the community (one asterisk); (b) somewhat representative of the average patients with NBNC-HCC in the community (one asterisk); (c) selected group of users, e.g., nurses, volunteers (no asterisk); and (d) no description of the derivation of the cohort (no asterisk). (2) Selection of the nonexposed cohort: (a) drawn from the same community as the exposed cohort (one asterisk), (b) drawn from a different source (no asterisk), and (c) no description of the derivation of the nonexposed cohort (no asterisk). (3) Ascertainment of exposure to (a) secure record (e.g., surgical records) (one asterisk), (b) structured interview (one asterisk), (c) written self-report (no asterisk), and (d) no description (no asterisk). (4) Demonstration that the outcome of interest was not present at the start of the study: (a) yes (one asterisk) and (b) no (no asterisk). ^‡^Comparability: (1) Comparability of cohorts on the basis of the design or analysis: (a) study controls for tumor stage (one asterisk) and (b) study controls for any additional factor (age, gender, tumor size, tumor location, etc.) (one asterisk). ^§^Outcome: (1) assessment of the outcome: (a) independent blind assessment (one asterisk), (b) record linkage (one asterisk), (c) self-report (no asterisk), and (d) no description (no asterisk). (2) Was the follow-up long enough for outcomes to occur: (a) yes (select an adequate follow-up period for the outcome of interest) (one asterisk) and (b) no (no asterisk). (3) Adequacy of the follow-up of cohorts: (a) complete follow-up (all subjects accounted) (one asterisk); (b) subjects lost to follow-up unlikely to introduce bias (small number lost), >80% follow-up, or description of those lost (one asterisk); (c) follow-up rate < 80% and no description of those lost (no asterisk); and (d) no statement (no asterisk).

**Supplementary Table 3 Subgroup analyses of survival outcomes between NBNC-HCC and B-HCC groups**

| Survival | Subgroup | No. of studies | Hazard Ratio | 95% CI | Test for overall effect | Heterogeneity | |
| --- | --- | --- | --- | --- | --- | --- | --- |
|  |  |  |  |  | *P* | *P* | I^2^ (%) |
| 5-year DFS | China | [23, 24, 41, 42, 45, 46] | 1.06 | 0.79, 1.41 | 0.71 | 0.003 | 72% |
|  | Japan | [9, 14, 15, 21, 30, 32, 33, 35, 36, 39, 40, 43] | 0.77 | 0.67, 0.88 | < 0.0001 | 0.14 | 30% |
|  | <100 cases | [9, 21, 26, 43] | 0.70 | 0.41, 1.18 | 0.18 | 0.49 | 0% |
|  | >100 cases | [14, 15, 23, 24, 30, 32-37, 39-42, 44-46] | 0.88 | 0.74, 1.05 | 0.17 | < 0.000001 | 80% |
| 10-year DFS | China | - | - | - | - | - | - |
|  | Japan | [14, 32, 33, 36, 37, 39, 40] | 0.84 | 0.69, 1.03 | 0.10 | 0.12 | 41% |
|  | <100 cases | - | - | - | - | - | - |
|  | >100 cases | [14, 32, 33, 36, 37, 39, 40] | 0.84 | 0.69, 1.03 | 0.10 | 0.12 | 41% |
| 5-year OS | China | [13, 23, 24, 29, 41, 45, 46] | 0.94 | 0.82, 1.08 | 0.41 | 0.18 | 33% |
|  | Japan | [9, 10, 14, 15, 22, 28, 30-34, 36, 37, 39, 40, 43, 44] | 0.94 | 0.88, 1.01 | 0.11 | 0.22 | 20% |
|  | <100 cases | [9-11, 22, 26, 28, 31, 43] | 0.98 | 0.88, 1.09 | 0.67 | 0.67 | 0% |
|  | >100 cases | [14, 15, 23, 24, 27, 29, 30, 32-34, 36, 37, 39-42, 44-46, 55] | 1.00 | 0.88, 1.13 | 0.96 | 0.08 | 34% |
| 10-year OS | China | [46] | - | - | - | - | - |
|  | Japan | [10, 14, 22, 28, 32, 33, 36, 37, 39, 40] | 0.93 | 0.79, 1.11 | 0.44 | 0.98 | 0% |
|  | <100 cases | [10, 22, 28] | 0.94 | 0.71, 1.24 | 0.64 | 0.69 | 0% |
|  | >100 cases | [14, 32, 33, 36, 37, 39, 40, 46] | 0.88 | 0.73, 1.06 | 0.17 | 0.90 | 0% |

NBNC-HCC, both HBV and HCV- negative HCC; B-HCC, HBV-positive and HCV-negative HCC; DFS, disease-free survival; OS, overall survival

**Supplementary Table 4 Subgroup analyses of survival outcomes between NBNC-HCC and C-HCC groups**

| Survival | Subgroup | No. of studies | Hazard Ratio | 95% CI | Test for overall effect | Heterogeneity | |
| --- | --- | --- | --- | --- | --- | --- | --- |
|  |  |  |  |  | *P* | *P* | I^2^ (%) |
| 5-year DFS | China | [23, 24] | 1.00 | 0.64, 1.56 | 0.99 | 0.35 | 0% |
|  | Japan | [9, 14, 15, 21, 30, 32, 33, 35-37, 39, 40, 43, 44] | 0.97 | 0.82, 1.16 | 0.75 | < 0.000001 | 80% |
|  | <100 cases | [24, 26, 43] | 1.13 | 0.75, 1.71 | 0.57 | 0.64 | 0% |
|  | >100 cases | [9, 14, 15, 21, 23, 30, 32, 33, 35-37, 39, 40, 44] | 0.96 | 0.81, 1.15 | 0.69 | < 0.000001 | 80% |
| 10-year DFS | China | - | - | - | - | - | - |
|  | Japan | [14, 32, 33, 36, 37, 39, 40] | 0.96 | 0.86, 1.07 | 0.49 | 0.26 | 22% |
|  | <100 cases | - | - | - | - | - | - |
|  | >100 cases | [14, 32, 33, 36, 37, 39, 40] | 0.96 | 0.86, 1.07 | 0.49 | 0.26 | 22% |
| 5-year OS | China | [23, 24, 29] | 0.82 | 0.33, 2.04 | 0.68 | 0.04 | 69% |
|  | Japan | [9, 10, 14, 15, 22, 28, 30-33, 36, 37, 39, 40, 43, 44] | 0.87 | 0.81, 0.93 | < 0.0001 | 0.0004 | 63% |
|  | <100 cases | [10, 22, 24, 26, 43] | 1.19 | 0.82, 1.74 | 0.36 | 0.76 | 0% |
|  | >100 cases | [9, 11, 14, 15, 23, 27-33, 36, 37, 39, 40, 44] | 1.07 | 0.87, 1.31 | 0.52 | < 0.0001 | 67% |
| 10-year OS | China | - | - | - | - | - | - |
|  | Japan | [10, 14, 28, 32, 33, 36, 37, 39, 40] | 1.00 | 0.87, 1.15 | 0.95 | 0.76 | 0% |
|  | <100 cases | [10] | - | - | - | - | - |
|  | >100 cases | [14, 27, 28, 32, 33, 36, 37, 39, 40] | 0.99 | 0.87, 1.13 | 0.90 | 0.76 | 0% |

NBNC-HCC, both HBV and HCV- negative HCC; C-HCC, HBV-negative and HCV-positive HCC; DFS, disease-free survival; OS, overall survival
